# Atomistic Simulation of Physical Vapor Deposition of Optical Thin Films

**DOI:** 10.3390/nano13111717

**Published:** 2023-05-24

**Authors:** Fedor Vasilievich Grigoriev, Vladimir Borisovich Sulimov

**Affiliations:** 1 Research Computing Center, M.V. Lomonosov Moscow State University (MSU), 119991 Moscow, Russia; v.sulimov@srcc.msu.ru; 2 Moscow Center for Fundamental and Applied Mathematics, 119991 Moscow, Russia

**Keywords:** optical coatings, thin film deposition, thin film properties, simulation of thin films, molecular dynamics, Monte Carlo

## Abstract

A review of the methods and results of atomistic modeling of the deposition of thin optical films and a calculation of their characteristics is presented. The simulation of various processes in a vacuum chamber, including target sputtering and the formation of film layers, is considered. Methods for calculating the structural, mechanical, optical, and electronic properties of thin optical films and film-forming materials are discussed. The application of these methods to studying the dependences of the characteristics of thin optical films on the main deposition parameters is considered. The simulation results are compared with experimental data.

## 1. Introduction

Optical coatings are widely used in various optical and optoelectronic devices, including mirrors, photovoltaic cells, filters, antireflecting coatings, photovoltaic cells, and so on [1]. Modern optical coatings consist of several dozen layers with alternating refractive indices. The number of layers, thickness, and material of each layer are determined by the purposes for which the coating is intended.

In film production, it is necessary to take into account the difference in the properties of films and bulk samples of the same chemical composition. For instance, the density of fused silica is equal to 2.2 g/cm^3^ [2], while the density of deposited SiO_2_ layers depends on the producing conditions and varies from 1.2 g/cm^3^ [3] to 2.4 g/cm^3^ [4]. The density of normally deposited films, when the atoms of the film-forming material approach the substrate almost perpendicular to it, is close to the density of bulk samples. At the same time the deposition under large angles results in the formation of highly porous anisotropic films with nanostructures having different dimensions and shapes [5]. The energy of the deposited atoms also significantly affects the structure of the film. High-energy deposition methods, such as ion-beam sputtering, produce denser and more uniform films than low-energy methods, such as thermal evaporation [1].

A detailed study of the dependencies of the film properties on the technological parameters of their production is important for improving the process of coating manufacturing. One of the tools for studying these dependencies is mathematical modeling of the growth of thin films and optical coatings. The most fundamental level of film formation modeling is atomistic. This level allows simulating the main technological conditions of film growth and calculates a wide range of their structural, mechanical, electronic, and optical parameters. The choice of a specific atomistic simulation scheme is determined by the technological method of film production. In this work, we focus on modeling the physical vapor deposition (PVD). This method has been intensively developed in recent decades and is used to produce multilayer coatings for a wide range of applications [1].

In this method, films grow in a solid substrate due to the condensation of matter from the gas phase [6,7,8]. The PVD process consists of several stages, which are characterized by various physical parameters and conditions, such as the energy of the particles involved in the process, temperature, composition of the target, the atmosphere in the vacuum chamber, and so on. For this reason, various methods are used to model these stages. In addition, the calculation of the structural, mechanical, optical, and electronic properties of optical films also requires the use of various methods of atomistic modeling, both classical and quantum.

In this work, we present a brief overview of the state of the art in atomistic modeling of the growth of thin optical films and the calculation of their characteristics. Special attention is paid to binary oxides that are widely used in the production of optical coatings: SiO_2_, TiO_2_, ZrO_2_, HfO_2_, and Al_2_O_3_. This article begins with a brief description of the PVD process and its stages. Next, we consider the methods used to simulate film growth and calculate film parameters. In the final part of the article, the simulation results are presented and discussed. Also, possible future directions of development in this area are discussed.

## 2. Main Stages of the PVD Process

**Figure 1:**
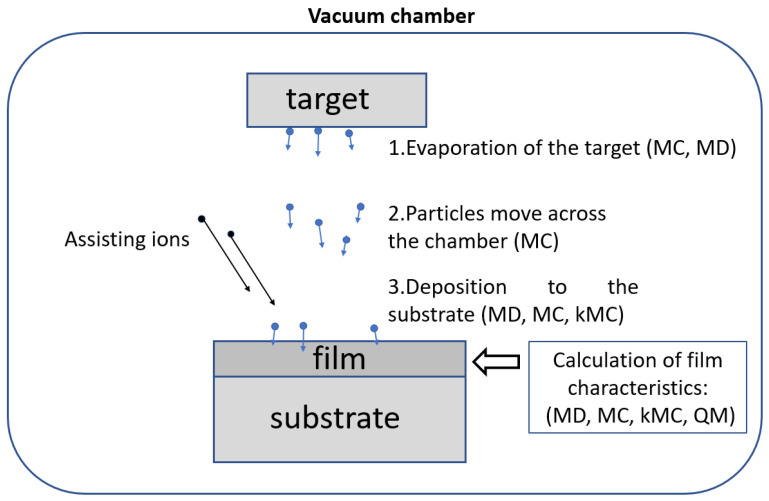
Scheme of the process of physical vapor deposition and methods used to simulate the stages of PVD: Monte Carlo (MC), molecular dynamics (MD), kinetic Monte Carlo (kMC), quantum methods (QM).

The PVD process consists of the following stages [1] (Figure 1):

1. Transfer of the target matter from the solid phase to the gas phase. Various methods are used for this: thermal evaporation, sputtering of the target substance with high-energy ions, reactive magnetron sputtering, electron-beam evaporation, etc.
2. Movement of particles from the target through the vacuum chamber to the substrate.
3. Deposition of particles on a substrate and formation of layers of growing films. High-energy assisting incident ions on the substrate can be used to improve the properties of the films.

As can be seen from Figure 1, MC-based methods are used at all stages of the PVD process. This is due to the fact that the MC approach can be effectively used in various physical conditions. The classical MD method is applied to simulate the condensation of incident particles on a substrate and the formation of film layers. The MD approach also allows calculating the structural and mechanical properties of the deposited film. Quantum methods (QM) are used only to calculate the properties of films and film-forming materials. This is due to the large computational costs of the QM compared to the classical methods.

Schemes for applying these methods to PVD process modeling are discussed in the next section.

## 3. Schemes of the Simulation of the Main Stages of the PVD Process

### 3.1. Brief Overview of Atomistic Modeling Methods

In the MD method, atoms in the simulation area move in agreement with Newton’s laws. Forces acting on atoms and the potential energy of interatomic interactions are determined by the force field. The force field includes a set of parameters, depending on the atom type, and a functional form describing the dependence of the potential energy on the interatomic distance. Interaction of the simulation area with the environment is determined by the boundary conditions. The fundamental aspects of the classical MD method are discussed in [9]. The result of the MD method is a simulation trajectory that includes the coordinates and velocities of all atoms in the simulation area. These data are used for the calculation of different structural and mechanical parameters of the deposited film. The MD simulation of thin film deposition can be performed using well-known program packets such as GROMACS (GROningen MAchine for Chemical Simulations) [10], LAMMPS (Large-scale Atomic/Molecular Massively Parallel Simulator) [11], and NAMD (NAnoscale Molecular Dynamics) [12]. Note that the LAMMPS [11] program has special functionality for the simulation of the deposition. Also, the LAMMPS provides the simulation with different types of force fields, including many-body and polarizable force fields. For these reasons, this program seems to be preferred for the simulation of the film deposition.

The Monte Carlo method has a similar thermodynamic basis as the MD method [13]. Accordingly to Metropolis scheme [14,15], the system transit from the initial microstate to the final one occurs in two cases: (1) if the difference of the energy of the final and initial state *E* < 0; and (2) if the *E* > 0 and exp(−*E*/*T*) > *r*, where *r* is the random number in the interval from 0 to 1, where *T* is the temperature of the system. The maximum change in the coordinates of particles during the transition depends on the interaction potential and the density of the system. This maximum change chosen so as the average ratio of the transitions to the attempts was not less than the specified value [15]. The force fields used in the MD method also can be used in MC simulation.

Kinetic Monte Carlo (kMC) is developed for the treatment of rare events in the simulated system [16]. The thermodynamic background of MD, MC, and kMC [17] are close, but kMC excludes the vibration movement of atoms. It allows modeling the long-time processes [18,19,20] with durations of 10^2^–10^3^ s for clusters including millions of atoms [21,22].

In the frame of the kMC, the film deposition is considered a sequence of the events such as adsorption of the incoming atoms to the empty site of the substrate, hopping of the atoms between sites, desorption, and so on. The probability *p* of the event depends on their activation energy *E*, temperature *T* and frequency *ν* of the attempts of the transition from the initial microstate to the final one:

*p*~ν∙exp(−*E*/*T*) (1)

As seen from Equation (1), the event probability is reduced exponentially with the growth of the activation energy. The diffusion of the atoms on the surface of the film and across the interlayer boundary is the example of a rare event with a large value of *E*. In [23], the activation energy of self-diffusion of Si in thermally grown SiO_2_ at temperature interval 1150–1300 °C is equal to 5 eV. Taking *ν*~10^14^·c^−1^ [24] and *T* = 1300 °C, we obtain *p*~4 × 10^−5^·c^−1^ that approximately corresponds to one event per second in the cluster consisting of 25,000 atoms. These rare events cannot be modeled using the usual MD and MC techniques.

The values of the activation energy can be taken from experimental data or calculated using the MD method [25,26] or other models [27,28,29,30]. Initially, this approach was applied to the growth of the crystalline films and was further extended to polycrystalline films [31,32]. It should be noted that, when using kMC to simulate the growth of amorphous films, difficulties arise due to the disorder of the structure. The variety of possible structural configurations requires the determination of a significant number of transition probabilities between them. In addition, many atoms can participate in such transitions, which makes it difficult to determine their probability. The kMC simulation of the film growth can be performed using the NASCAM (NAnoSCAle Modeling) program [21], which was developed for this goal. The combined MC-MD and kMC-MD methods are also used in the simulation of the thin film deposition [33,34].

The quantum methods represent the most fundamental level of the atomistic simulation. The high computational cost of these methods limits the number of atoms in the simulation clusters to a few hundred, so QMs are not directly applied for the simulation of the deposition process. At the same time QMs can be used for the simulation of the point defects of different types, electronic, and optical properties of deposited films. The initial geometry for the quantum simulation can be taken from the clusters, obtained by the classical methods such as MD and MC. The QM also used for the parametrization of the force fields for the MD, MC, and kMC methods. There are a lot of programs for QM modeling that can be used for the mentioned goals, in particular, CRYSTAL [35]—optimization of the geometry of the ground state with periodic boundary conditions, VASP (Vienna Ab initio Simulation Package) [36]—quantum MD with periodic boundary conditions, and the GAMESS (General Atomic and Molecular Electronic Structure System) [37] cluster approach.

### 3.2. Sputtering of the Target

The structure and properties of the growing films significantly depend on the energy of the particles arriving to the substrate. In low-energy methods (thermal evaporation, electron-beam evaporation) the parameters of the flow of the emitted particles from the target are defined using the continual methods [38]. For instance, the mass flow rate is defined using the Hertz–Knudsen theory [39]. In the high-energy PVD processes, atoms are sputtered from the target by the beam of ions, accelerated by the electric field. The energy of the ions can achieve several keV [40]. The description of the process of the interaction of these ions with target matter requires both continuum and atomistic methods.

**Figure 2:**
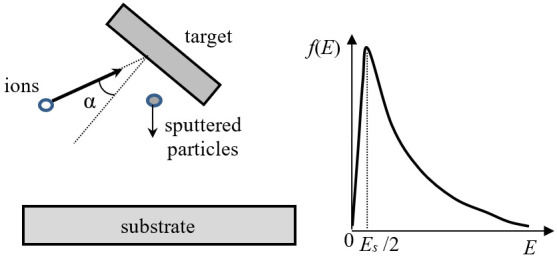
Geometry of the sputtering process, α is the incident angle (**left**) and schematic representation of the energy distribution of the sputtered particles using the Thomson formula [41] (**right**). The distribution maximum is located about of *E_s_*/2, where *E_s_* is the surface energy of the target atom (~several eV).

The simplified scheme of the sputtering process is shown in Figure 2. The input parameters for modeling are the energy and mass of the bombarding ions, the angle of incidence, and the ion flux density. The characteristics of the target substance are also taken into account: the surface energy of the target atom and the potential of interatomic interactions inside the target and between high-energy ions and the target atoms. Usually, the following characteristics of the sputtering process are calculated: sputtering coefficient, i.e., the ratio of the number of sputtered particles and incident ions, angular and energy distributions of the particles, and distribution of particles by the charge and number of atoms.

A Monte Carlo simulation of the binary collisions is one of the most common methods applied to study sputtering. In the frame of this method, it is assumed that the incoming ion collides with one target atom at a time [7]. A high-energy ion loses energy due to collisions with the target atoms. Also, the inelastic energy losses are taken into account in the frame of the different approaches, depending on the energy of the ion [42,43,44].

In [45], the Monte Carlo method, implemented in TRIM (TRansport of Ions in Matter) program [46], was proposed to obtain the energy and angular distributions of the sputtered particles. The target is considered amorphous, so the atoms are distributed randomly. The effects of the energy of interatomic interactions and the surface binding energy on the deposition process were considered. The authors concluded that the proposed method reproduces experimental results related to the energy and angular distributions of sputtered particles. The method, proposed in [45,46] was further developed in the frame of the SRIM (The Stopping and Range of Ions in Matter) program [47], which includes the database of target materials and electronic energy loss.

The experimental investigation and Monte Carlo simulations of the ion sputtering of the germanium target was performed in [48]. In the experiment, the energy of the argon and nitrogen ions is equal to 20 keV, and incident angle varies in the range of 60–85°. It was established that the initial simulation results differ significantly from the experimental ones. Correction of the model of ion penetration into the target leads to better agreement between simulation and experiment.

In [49], the possibilities and limitations of three popular programs (SDTrimSP [50], TRIDYN [51], and SRIM [47]) for the Monte Carlo simulation of sputtering were discussed. The authors focused on the calculation of the different aspects of the sputter yield and angular distribution of sputtered particles. It was found that the experimental dependence of the sputter yield on the ion incident angle is well reproduced by the SDTrimSP program in the case of 1 keV Xe ions for the Si and Ge targets. The authors of [49] concluded that SDTrimSP allows calculating a wide range of parameters describing the sputtering process, including sputtering yield and angular and energy distribution of particles. In addition, the effects related to the sputter yield amplification can be studied. The SDTrimSP program allows simulating the sputtering of composite targets. TRIDYN has similar functionality, but fewer options for the input and output of data describing the conditions and results of the sputtering process. The results of the sputtering simulations obtained by SDTrimSP and TRIDYN agree with the experimental data.

Classical MD also was used for the simulation of the sputtering. In [52], the results of MD simulation obtained by the end of the nineties was reviewed. It was noted in [52] that the MD can provide the data that are difficult to obtain by other methods: cluster emission, acting of the sputtering to the surface relief of the target, and chemical effects. In particular, the simulation results show that when the Ag target is bombarded by 5 keV Ar ions, the portion of the sputtered cluster exponentially reduces with the growth of the number of atoms in the clusters. The simulation results agree with experiments in which the observed fraction of dimers during sputtering of metal targets is about 10% [53].

The MD simulations of sputtering of Cu (111) surfaces by Cu and Ar ions were performed in [54]. The dependence of the sputter yield, average emission angle of the sputtered particles, and other characteristics on the incident angle and energy of ions were obtained. It was found that the calculated sputter yields in the case of the normal incidence of the ions are in agreement with the experiment.

In [55], the energy distributions of the platinum atoms sputtered by argon ions with energies 0.1, 0.5, and 1.0 keV were obtained using the MD simulation. It was found that these distributions are close to those given by the Thompson formula [41] based on the theory of binary collisions [56]. In [57], the multi-time scale approach was used to simulate the sputtering of gold targets by Au and Ar ions with energy 0.5 keV. The fast process of the collisions of the high-energy ions with the target was simulated by the MD method, while the kinetic Monte Carlo method [58,59] was used to simulate the relative long-time relaxation and diffusion processes. The Ackland EAM (Embedded Atom Model) potential [60] for Au was used, but for the close interactions, it was replaced by the screened Coulomb ZBL (Ziegler, Biersack and Litt- mark) potential [47]. The crystalline recovery of the Au target was observed between impacts in [57]. It was found that the energy distributions of the sputtered atoms are in good agreement with those experimentally measured.

In [61], the MD simulation of the sputtering of the crystalline Si target by the Ar ions was performed. The interactions between silicon atoms were described using the Modified Embedded Atom Method (MEAM) potential and the Stillinger–Weber potential. It was found that the difference in the interatomic potentials results in the essential difference in the sputter behavior. Additionally, the DFT calculations revealed the shortcomings of both potentials in the description of the interatomic interaction. For this reason, the MEAM potential was refit based on the DFT calculations.

MD simulation of the ions bombardment of the amorphous silicon oxide and Si_3_N_4_ targets was carried out in [62]. The energies of the Ar+ ions were taken equal to 100 and 200 eV, and the incident angle varied from 30° to 85° with a step of 15°. It was found that the sputtering yield of Si_3_N_4_ target was more than twice that of the SiO_2_ target at the investigated interval of the sputtering parameters.

Analytic models were suggested for calculating the distribution of the sputtered clusters on the number of atoms [63,64,65]. The mean values of the number of the atoms are calculated for the niobium, tantalum, silver, and iron targets. In particular, the values of the average energy of the sputtered clusters are in the interval of 4–5 eV, which agrees with the experiments [66,67].

In the recent review [68], the theoretical basis of the sputtering process and recent models were described, focusing in particular on the energy distribution of the sputtered particles. Applying such atomistic methods as MD, MC, and kMC to the simulation of the interaction of the high-energy ions with target and the transport of the sputtered particles to the substrate and its deposition was considered in [68].

Thus, in recent decades, different methods of the atomistic level were applied to study the sputtering process and dependencies of the parameters of the sputtering particles on the characteristics of the targets and incoming high-energy ions. The complexity of the task is due to the nonequilibrium character of the sputtering process and the wide interval of the energy of the interatomic interactions. The last condition requires applying the different methods for the adequate description of the energy transfer from the incoming ions to the target and the further redistribution of this energy between particles of the target. The evaporation of the atoms and molecules and emission of the electrons from the target are also complex processes requiring a description at the quantum level. Thus, the predictive simulation of the sputtering, describing, in particular, the distribution of the emitted particles on the charge and number of the atoms, is still a challenging task.

### 3.3. Movement of Particles toward the Substrate

The particles, emitted from the target, travel to the substrate across the vacuum chamber. The energy and angular distributions of the emitted particles can change due to collisions with the atoms and molecules of the gas in the vacuum chamber. The average distance between two collisions depends on the chamber gas pressure and temperature, particles radii, energy of the emitted particles, and other parameters [69]. In the case of the thermal evaporation of the target, the collisions of the emitted particles with gas atoms are rare due to low pressure (~10^−4^ Pa) [70]. As the pressure increases, the collisions should be considered in the simulations. The loss of the kinetic energy of the sputtered particles and change in the direction of their movement can be quantitatively taken into account using the expressions given, for example, in [69].

The influence of these collisions on the energy and angle distributions of the sputtered particles can be considered in the frame of the Monte Carlo method. The simulation of the transport of atoms from the target to the substrate was performed in [71] using the MC program SIMTRA (Simulation of Metal TRAnsport). The initial energies of the sputtered atoms in this program can be specified using the Thompson distribution. The erosion profile of the target also can be taken into account. The vacuum chamber geometry was implemented into the code, and its influence on the deposition process was discussed. In [72], the continuum-generalized multiphase-field model predicting the growth of polycrystalline thin films, fabricated by PVD, was developed. The input parameters for the model were partly obtained from the MC simulation with SIMTRA. The magnetron sputtering conditions were applied to obtain the energy and angular distributions of the arriving to substrate atoms. The simulation profiles of growing films predicted by the simulation are in agreement with the experimental data. In [73], the complex model of the reactive magnetron sputtering was proposed. The model describes the wide range of processes affecting the structure of the deposited films, including reactive gas chemisorption, implantation of reactive species in the target, deposition of the sputtered particles to the substrate, and reverse process. The proposed model is developed using the facilities of RSD2013 (Reactive Sputter Deposition) software [73], which was previously developed for modeling of the reactive sputter process. In a recent review [74], the application of the different simulation methods, both continuum and atomistic, for studying the magnetron sputtering discharge was discussed. It was noted that the MC method is useful for studying the transport of the particles and collisions processes in magnetron sputtering discharges. The direct MC method [75] was applied for the prediction of the ion trajectories, energy and angular distributions of film-forming particles, and their thermalization. The particle-in-cell/Monte Carlo (PIC/MCC) method and its application to study the magnetron sputtering discharge was considered in [74]. This method is close to the direct MC method, but its advantage is that the distribution of the electric field calculated self-consistently using the Poisson equation from the positions of the charged species. In [76], the application of the PIC/MCC method for the simulation of the magnetron sputtering discharges was discussed in detail.

In [77], the direct MC method, utilizing the solving the Boltzmann equation, was applied to simulate the transport of the neutral particles in a vacuum chamber. The ion beam sputtering process was modeled taking into account the real geometry of the coater. The energy and angular distributions obtained in this simulation were further used in kMC and MD simulations of film growth.

Thus, the tools and possibilities for modeling particles emitted from a target depend significantly on the technological conditions of the PVD process. In the case of low-energy methods, such as low-pressure thermal evaporation, the emitted particles move from the target to the substrate in an almost straight line, since the probability of collision is low. On the other hand, transport modeling in the case of high-energy methods such as ion-beam sputtering is difficult due to the relatively high gas pressure in the vacuum chamber and the more complex angular and energy distributions of the sputtered particles. The need to take into account electric and magnetic fields in the deposition process complicates the simulation. Methods based on MC in various implementations are commonly used to model the transport of emitted particles.

### 3.4. Simulation of Thin Film Deposition

The films are formed by the atoms emitted from the target and deposited onto the substrate. Growth of the films is accompanied by the oxidation of the incoming atoms, forming the chemical bonds between these atoms and matter of the earlier deposited layers, the surface diffusion of the atoms. These processes affect the structure and properties of the film and should be modeled at the atomistic level using methods such as classical MD, MC, and kMC. The simulation schemes are similar for all of these methods, so we will describe them for the MD case with additional explanations as needed.

#### 3.4.1. Scheme of the Simulation

**Figure 3:**
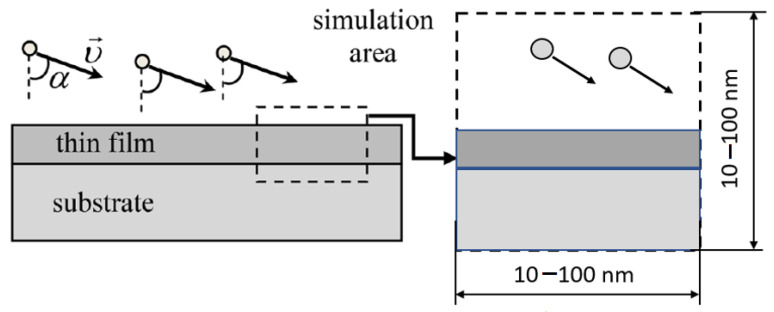
Choice of the MD simulation area, α is the deposition angle, υ is the velocity of the incoming atoms. Reprinted from ref. [78].

The MD simulations are performed at atomistic clusters with dimensions 10–100 nm, representing the growing film (Figure 3). The concrete value of the cluster dimensions strongly depends on the force fields used in the simulation (see next Section 3.4.2 for the details). The periodic boundary conditions are applied in both horizontal directions. In the vertical direction, the rigid wall usually restricts the movement of the atoms.

**Figure 4:**
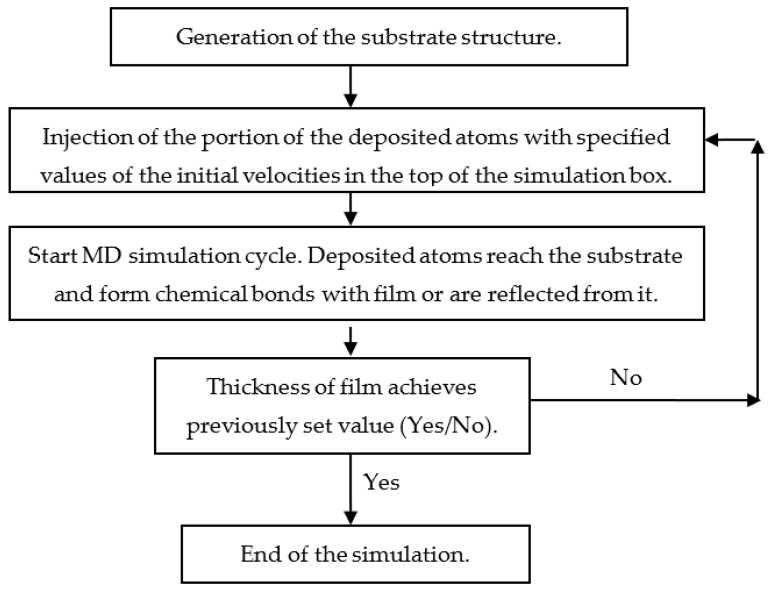
Scheme of MD simulation of the thin film deposition.

Film growth simulation is organized as a step-by-step procedure (Figure 4). Before starting the simulation of the deposition, it is necessary to prepare the atomistic structure of the substrate. Such a structure can be obtained, for example, using a melt-quenching procedure [79].

When the substrate is prepared, the main part of the simulation procedure, consisting of the sequence of the same MD cycles, starts (Figure 4). The duration of the MD cycle is about several picoseconds [34,80]. The initial coordinates of the injected atoms are specified randomly, and the values and directions of their initial velocities correspond to the specified energy and angular distributions of the deposited atoms. During every MD cycle, the atoms injected in the top of the simulation box move to the substrate and form the chemical bonds of the surface of the growing film or are reflected from it. According to experimental conditions, reflected and resputtered atoms are removed from the simulation box. The number of MD cycles depends on the specified thickness of the deposited film.

The following technological parameters, acting as the film structure and properties, are taken into account in this scheme:

1. Energy distribution of the incoming atoms is determined experimentally or calculated from the simulation of the target evaporation and transport of particles. In MD simulation the energy distribution is specified in terms of the initial values of the kinetic energy of injected atoms.
2. Deposition angle (see Figure 3). In the experiments, usually the dimension of the substrate is much smaller than the average distance between the target and substrate, so the velocity direction for all incoming atoms can be considered as the same. If this condition is not satisfied, one can set the angular distribution in terms of the initial velocities of the atoms.
3. Experimental value of the substrate temperature can be set and kept constant during simulation using thermostat—special computational procedure developed for this goal in MD [81]. It should be noted that using one of the most popular Berendsen thermostats on the trajectory with length~tens nanoseconds can lead to the wrong conclusions [82]. The temperature of the system in MC is specified directly in the expression of the probability of the transition of the system from the initial microstate to the final one.
4. The atmosphere of the vacuum chamber can be taken into account by introducing molecules into the simulation area in an amount corresponding to the gas concentration. The temperature of the atmosphere is specified by the velocity distribution of molecules.
5. Parameters of the assisting ions flow, if used: composition, energy, and angular distribution. These parameters are set in the same way as for the deposited particles.
6. External pressure, if it is applied to the substrate. Pressure is set using the MD procedure, termed barostat [81].
7. External electric field.

It should be noted that the typical film growth rates are ~nm/s [1], so approximately one molecule per millisecond is deposited onto a substrate element with dimensions of 10 nm × 10 nm. While the characteristic simulation times using the traditional MD technique are on the order of nanoseconds, using hyperdynamic techniques [83], the characteristic times can be increased to microseconds and even milliseconds [84], but this acceleration is not enough to simulate film deposition at an experimental rate. Thus, the growth rate in the MD simulation is many times higher than the real one. In fact, the MD method makes it possible to consider only short-term processes with a duration of no more than picoseconds, such as the transfer of kinetic energy from deposited atoms to films, the formation of bonds on the surface, and thermal relaxation near the collision point of high-energy ions with film [85]. To model long-term processes such as surface diffusion, methods such as kMC should be used.

The described scheme can be part of a more general multiscale modeling scheme that takes into account all stages of PVD, as suggested, for example, in [86]. The movement of the sputtered particles through the chamber was simulated by the 3D direct Monte Carlo method. The kMC approach was used to simulate film growth on a substrate. The angular and energy distributions of particles arriving at the substrate were taken from the results of modeling their motion across the chamber.

#### 3.4.2. Choice of Force Field

The choice of a force field that describes the potential energy of interatomic interaction is one of the key points in classical atomistic modeling. Relatively simple pairwise force fields [87,88,89], which are also popular in the modeling of the biomolecules [90], are often used for the simulation of thin film deposition. In these force fields the expression for the potential energy consists of two terms, describing electrostatic and Van der Waals contributions. The last term takes into account the short-range repulsive exchange interaction, as well as the long-range attractive dispersion and polarization interactions. As a rule, the electrostatic contribution is considered as the interaction of the point charges centered in the atoms. The van der Waals interaction energy is usually calculated using the Morse potential [87,88,91,92,93] and Lennard–Jones potential [94,95]. The pairwise force fields have high numerical efficiency, which makes it possible to simulate clusters of thin films with characteristic sizes of several tens of nanometers. In [96], the study of the different force fields for the description of ZrO_2_ film growth was performed using the MD method. The force fields parameters that ensure the experimentally relevant structures in MD simulation were found. In [97], a similar study was performed for TiO_2_ films. For the MD simulation of the HfO_2_ films, the Born–Mayer–Buckingham potential was used in [98] with the parameterization described in [99]. The pairwise force field with Buckingham-type interatomic potential was used in the MD simulation of ZnO [100]. The new method of the parametrization of the force fields for the classical atomistic simulation of such matter as oxide glasses was proposed in [101]. For the fitting of the force field parameters, the radial distribution function, vibrational density of states, and dependence of the glass density on pressure were used. Also in [102], the new empirical force field for ionic and semi-ionic oxides was suggested. The reliability of the force field was demonstrated by testing it relative to the prediction of the structural and mechanical properties of a wide range of silicates.

Many-body and polarizable force fields [103,104,105,106] were also used for the simulation of the deposition process. The REaxFF (REActive Force-Field) force field based on the parametrization of the quantum density functional (DFT) calculations [107] and having the functional form allowing describing the formation of the chemical bonds, provides the perspective for the simulation of the film growth. In the last decade, the new class of force fields based on machine learning was developed (see, for instance, the review [108]). These force fields include many parameters that are fitted to reproduce the experimental properties of the matter and results of the quantum simulations. The complicated functional form of the polarizable, many-body, and machine-learned force fields essentially limits the dimensions of the simulation clusters, while methods are being developed to speed up calculations with these force fields [109].

The specific features of the abovementioned types of force fields are summarized in Table 1. The study of large-scale inhomogeneities in the film, like pores, columnar structures, calculation of the roughness, and mechanical stresses, requires large clusters with characteristic dimensions of several tens of nanometers. Modeling of these clusters should be performed using the pairwise force fields with a simple functional form and having high computational efficiency. To study the formation of the complicated point defects, arising, for instance, due to bombarding of the substrate by high-energy incoming atoms, the more sophisticated many-body force fields like REaxFF are required. Also, the implementation of variable charges in REaxFF makes it possible to describe non-stoichiometric compositions using a single potential.

#### 3.4.3. Technical Aspects of Simulation Procedure

One of the key parameters affecting the computational efficiency of MD simulation is the time step of numerical integration of the equation of motion. Usually the default value is *dt* = 1 fs [15]. This value of *dt* can be taken when modeling low-energy deposition with kinetic energy of incident atoms is about *E* = 0.1 eV [70]. At the same time, simulation of high-energy deposition with *E*~1–10 eV may require a decrease in the value of *dt*. The accuracy of the simulation depends on the displacement of the atom per time step, *s* = *vdt*. An increase in the kinetic energy of the incident atoms by *n* times leads to an increase in the velocity by *n*^1/2^ times. Thus, if we keep the value of *s* constant, the value of *dt* must decrease by a factor of *n*^1/2^. Accordingly, the simulation time increases by *n*^1/2^ times.

To reduce the increase in simulation time, for the case of high-energy deposition, the MD cycle can be divided into two stages [110]. The first stage describes the energy transfer of incident atoms to the substrate and films. The duration of this stage is about 2 ps [80], which is enough for the high-energy atoms to lose their energy. The *dt* value in this stage should be reduced compared to 1 fs accordingly to the deposition energy. The second stage has a usual duration 6 ps and *dt* value of 1 fs.

As mentioned in the previous section, the functional form of the force field significantly affects the simulation time. It is important that transparent films, as a rule, consist of at least two components, which leads to the appearance of partial charges in atoms. So, for a correct description of interatomic interactions, an electrostatic term in force fields is needed. The calculation of this term is the most time-consuming procedure in MD simulations. Therefore, in the atomistic simulation of transparent media, the number of atoms is much smaller than in the simulation of one-component media, such as metals [11]. The method of the truncation of the potential, which is used for the Van der Waals interactions, in this case results in the artifacts of the simulation results (see, for instance, [111,112]). For this reason, the energy of electrostatic interaction in the MD simulation of transparent media is calculated without truncation. Usually, the Particle Mesh Ewald (PME) [113] method, approximating the Ewald sum [15] of the pair contributions when the periodic boundary conditions are applied to the system, is used in MD simulations. This method scales as *N*ln(*N*), where *N*—number of atoms in the simulation clusters. The number of atoms grow linearly with the number of MD deposition cycles. So, the simulation time of the deposition process grows as the square of the number of deposited atoms, if we neglect the logarithmic multiplier.

To simulate nanoscale structural peculiarities in films, the clusters with dimensions of approximately tens of nanometers and consisting of millions of atoms are required. In these cases, the parallel computations should be used to reduce the simulation time. All the well-known programs for the MD simulation, such as GROMACS [10], LAMMPS [11], and NAMD [12], have the corresponding functionality. The efficiency *f* of the parallel computations with GROMACS was estimated in [114]. The value of *f* was calculated as *f*(*N*) = (32/*N*) × *T*(32)/*T*(*N*), where *N* is the number of computational cores. The simulation was carried out using the equipment of the shared research facilities of HPC computing resources at Lomonosov Moscow State University [115]. The cores of Intel Haswell-EP E5-2697v3, 2.6 GHz processors were used in the computations. The pairwise DESIL [80] force field was used for the calculation of the potential energy of the interatomic interactions. The wall time of the deposition of 2 × 10^6^ atoms using 128 cores was about four days with *dt* = 1 fs and duration of one MD cycle 6 ps. As is shown in [114], the *f* value monotonically decreased with the increase in the number of the computational cores *N*, and was equal to 0.4 at *N* = 512.

## 4. Results of the Simulation of Thin Optical Films and Film-Forming Materials

### 4.1. Structural Properties

The results of full-atomistic modeling of the structure and properties of growing films essentially depend on the force fields that describe interatomic interactions. To validate these force fields, the properties of film-forming materials are calculated and compared with experimental data. The results of the calculations of the density of silicon dioxide and titanium dioxide using different force fields are presented in Table 2. The many-body polarizable force field ReaxFF [116], based on the fitting of the quantum chemistry simulations, reproduces well the experimental value of the fused silica density ρ_g_ = 2.2 g/cm^3^ [2]. The nonpolarizable pairwise force fields, such as DESIL (DEposited SILica) [117] and others [87,118], also reproduce ρ_g_ value. The ab initio-based BKS force field (Beest Kramer van Santen) [89] essentially overestimates the ρ_g_ value. The many-body Tersoff potential [104] underestimates the density of the silica polymorphs (α-Quartz, β-Quartz, α-Cristobalite, β-Cristobalite) approximately by 0.1–0.2 g/cm^3^ and overestimates the ρ_g_ value.

The pairwise force fields allow reproducing the density of the titanium dioxide. The MA force field [119], consisting of Coulomb, dispersion, and repulsion terms, reproduces well the geometry of the elementary cell of TiO_2_ polymorphs and their density. The force field with Coulomb and Lennard-Jones terms [120] was used in [95] to simulate the structure of titanium dioxide films. The density of the rutile and amorphous TiO_2_ was reproduced in [95]. The partial-charge Buckingham interaction potential was developed in [97] especially for the atom-by-atom simulation of the film deposition. The potential reproduces the density of the amorphous TiO_2_ (Table 2).

**Table 2:** Calculated using of different force field, ab initio quantum methods (*ab*) and experimental (exp.) values of density, ρ (g/cm^3^).

SiO_2_									TiO_2_		
									Rutile	Amorphous	
ρ	2.2 (exp.) [2]	2.18 [116]	2.21 [87]	2.58 [89]	2.3 [121]	2.2 [118]	2.42 [104]	2.14 [117]	4.3 (exp.) [122] 4.3 [95] 4.3 [119]	3.8 (*ab*) [123] 3.9 (exp) [124] 3.8 [95]	3.9 [97]

The refractive index of optical film is density dependent, so reproducing this structural characteristic is an important simulation test. Results of the density calculations using atomistic simulation methods are presented in Table 3. In [34], using the polarizable Tersoff potential, the dependence of the SiO_2_ film density on the energy of incident atoms was studied. The energy of 5 eV and 10 eV corresponds to high-energy deposition methods like ion beam sputtering, and 0.1 eV corresponds to low-energy methods like thermal evaporation [125]. It was shown that the increase in the energy of the deposited atoms results in an increase of the film density. This tendency agrees with the experimental data [1]. The refractive index of the high-energy deposited SiO_2_ films is larger than that of fused silica [126], therefore, the density of these films should exceed the fused silica density 2.2 g/cm^3^. This condition is satisfied by the results of the MD simulation of the deposition process using a paired force field DESIL [80] consisting of Coulomb and Lennard–Jones terms (see Table 3, [85]). The difference in the density of high-energy deposited film and fused silica results in the respective difference of refractive indices, which can be estimated using the Gladstone–Dale equation [127] in the form Δn = 0.2Δ = 0.04, which corresponds to the experimental results [128].

The MD simulation of the TiO_2_ films using the pairwise MA (Matsui and Akaogi) potential [119] was performed in [77,129]. The low-energy deposition produces porous film with low density. The increase of the energy of the deposited atoms results in an increase in film density. The similar tendency was observed in [95], but the difference in the density for low- and high-energy deposition, obtained in [129], is noticeably larger than in [95]. This can be explained by the relatively low dimensions of the simulation clusters used in [129]. The experimental value of the density of the low-energy-deposited TiO_2_ films varies from 3.0 g/cm^3^ to 3.6 g/cm^3^ and essentially depends on the pressure in the vacuum chamber [130].

**Table 3:** Density ρ (g/cm^3^) of silicon dioxide and titanium dioxide thin films, E(eV)—energy of deposited silicon atoms.

SiO_2_						TiO_2_		
E	0.1	1	5	10	Exp.	0.1	1	10
ρ	2.06 [131]	1.95 [34] 2.29 [85]	1.98 [34]	2.1 [34] 2.41 [85]	1.95 ^1^ [132]	3.5 [95] 2.75 [129] 3.4 [77]	3.85 [129]	3.9 [95] 4.15 [129] 4.15 [77]

^1^ Film is produced by the electron beam evaporation method (low-energy deposition).

**Figure 5:**
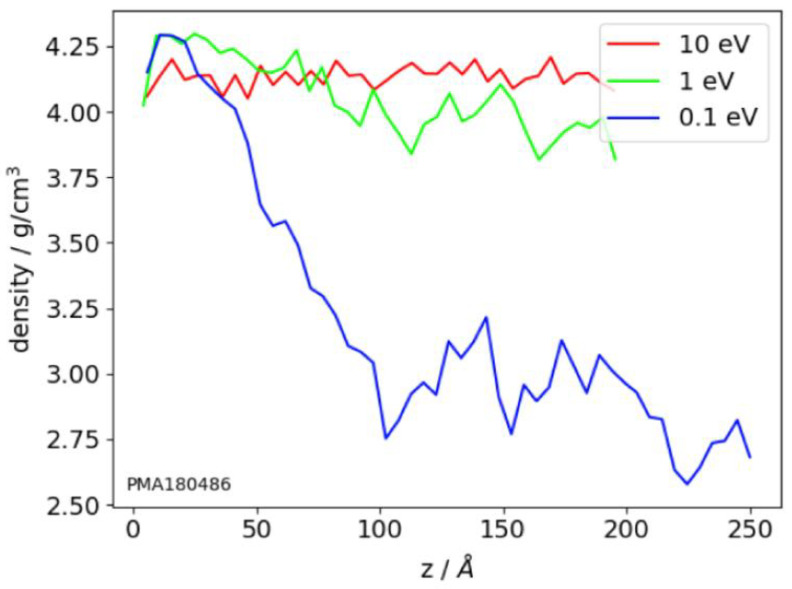
Density profiles of the TiO_2_ films, z is the vertical coordinate of the films layer. Energies of the deposited Ti atoms are shown. Substrate temperature is equal to 300 K. Reprinted from ref. [129].

The density profiles, calculated based on the structure of the atomistic clusters, show density distribution across the film thickness [80,95,129,131]. To calculate the profiles, the film volume is divided into layers, parallel to the substate plane, and further density is calculated for every layer. For high-energy films, these profiles are smoother than for low-energy films (Figure 5). The large fluctuations in the density profiles indicate the high porosity of the films. Also, the density profiles show the thickness of the transition layers between substrate and film and film and vacuum. This value is about of 2 nm thickness [80,95].

The radial distribution function (RDF), showing the average distance between atoms, can also be calculated based on the results of the atomistic simulation. In [34], the RDF was calculated for SiO_2_ films obtained by the MD simulation with many-body potential of the Stillinger–Weber type [103]. The authors of [34] conclude that the calculated RDF corresponds to the amorphous state of silicon dioxide. The RDF was calculated in [104] for silicon dioxide clusters obtained by the MD simulation with modified Tersoff potential [133]. It was found that the positions of the first and seconds peaks are in good agreement with the experiment [134] and results of the ab initio MD simulation [135]. At the same time, the second peak of calculated RDF was sharper than that of the experimental RDF. In [136], the different force fields for amorphous silica were studied using the atomistic MC method. It was found that all investigated potentials have the same positions of the RDF peaks. In [137], the MD simulation of SiO_2_ thin films was performed using the ReaxFF potential. The structure of the film was analyzed using the RDF. It was found that the film structure in the surface region differs from the structure of the interior area.

In [138], the RDF’s for fused silica and high-energy deposited films were calculated and compared. The atomistic clusters were obtained using the MD method with a pairwise force field [80]. It was found that the peak positions, corresponding to the Si-O bonds and minimal O-O and Si-Si distance, coincide for films and fused silica. Taking this into account, the excess film density compared with fused silica was explained by the difference in the mutual orientation of the structural tetrahedrons [138]. A similar approach was used to calculate the RDF of TiO_2_ films [95]. It was found that the first Ti-O peak near 2.0 nm corresponds to the length of the chemical bond between titanium and oxygen atoms in amorphous TiO_2_ [139]. The positions of the first peaks on Ti-Ti and O-O RDF’s also coincide with the experimental data [139] and previous simulation results [105,140]. It was concluded in [95] that the force field, proposed in [120], reproduces the structural characteristics of amorphous TiO_2_ and can be applied for the simulation of the structure of TiO_2_ films.

The surface roughness *R_h_* can be calculated as the root square deviation of the vertical coordinates of the surface atoms [141]. The *R_h_* value for silicon dioxide films was calculated in [79,142] using the MD method with a pairwise force field. The calculated values of *R_h_* were in agreement with the experiment [143]. The simulation showed that a decrease in the substrate temperature leads to an increase in *R_h_* under conditions of low-energy deposition. An increase in the energy of the incident atoms leads to a decrease in the surface roughness. The dependence of the *R_h_* on the density of the assisting ions flow *f* in the MD simulation of the silicon dioxide film deposition was studied in [110]. It was found that the increase of *f* from 4% of density of the deposited atoms flow to 10% results in the decrease of *R_h_* from 0.57 nm to 0.34 nm. Further increase of the *f* value acts insignificantly on the surface roughness.

The *R_h_* value of TiO_2_ films deposited using the kMC method was calculated in [77]. An increase of *R_h_* with an increase in the number of the deposited atoms in the initial stage of the film growth was demonstrated. The maximum value of *R_h_* is equal to 1.5 nm, when the number of deposited atoms achieves 2 × 10^6^. Also, the *R_h_* increases with growth of the deposition angle. An increase in the energy of the incoming atoms from 0.1 eV to 10 eV results in a reduction of the *R_h_* from 1.3 nm to 0.2 nm if the deposition angle equals zero. The roughness of the TiO_2_ films was calculated in [95] using MD simulation. The films, deposited at energy 10 eV (high-energy deposition) have a roughness of less than 0.2 nm. The decrease in the *E* to 0.1 eV (low-energy deposition) leads to an increase of *R_h_* to 0.8 nm.

The porosity of thin films is of great importance, since the pores can contain small molecules affecting the optical properties of the films. The porosity of the titanium dioxide films was studied in [22,77]. The film cluster consisting of two million atoms was deposited using the kMC method with pairwise potential. It was found that at low-energy deposition, the total volume of the pores with diameters of more than two atomic dimensions is about 7% of the film volume. The dependence of SiO_2_ porosity on the deposition condition was studied in [144] using the MD method. The relative pore volume function *f*(*R*), depending on the effective pore size *R* was calculated in [144] (Table 4). An increase in the deposition energy leads to a decrease in the relative volume of pores, especially for large pores. The existence of the pores with dimensions ~ 1 nm in the low-energy deposited films was observed experimentally [132].

Similar results were obtained for titanium dioxide films using the same method [95].

The formation of the point defects in the SiO_2_ films was studied using MD methods in [142]. It was revealed that the main point defects are nonbridging oxygen O_1_ (the low index is the coordination number) and overcoordinated oxygen atom O_3_. The concentration of the point defects increases with decrease of the energy of the deposited atoms. The annealing of the SiO_2_ results in a noticeable reduction of the concentration of all types of defects.

Rings consisting of different numbers of atoms *n* (*n*-membered rings) are formed in a lot of film-forming compounds. The distribution of rings over the number of atoms is used to characterize the structure of the film. For example, a high concentration of rings at a small n indicates the presence of internal stresses in the substance [145]. The distribution is calculated using the special methods [146,147], which use the cartesian coordinates of the atoms and number and length of the chemical bonds that an atom forms with neighboring ones. Based on the analysis of the SiO_2_ clusters, obtained from MD simulation of fused silica, the ring distributions were calculated in [104,145,148,149]. The maximum of the distributions was observed for the *n* = 6. The distributions are close to symmetrical around the central peak, and rings with *n* < 3 and *n* > 9 are not observed in [104,145]. In [148], the distribution maintains symmetry only for the peaks closest to the central one. Similar distributions were obtained in [101]. The distributions described above agree with the experimental results for the fused silica [150,151]. The distributions of rings in SiO_2_ obtained using the difference force fields were compared in [136]. For all the force fields, the peak of the distributions corresponds to the six-membered rings. It was also found that the relative contribution of the strained rings with a number of atoms less than five is minimal for the Tersoff potential [104].

**Figure 6:**
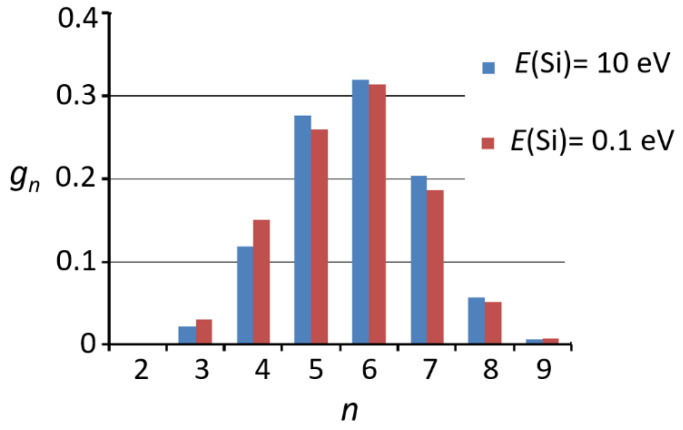
The relative concentration *g_n_* on the rings with different number of atoms *n* in SiO_2_ films deposited at different value of the deposition energy *E*. Adapted from ref. [152].

The distributions of the rings in silicon dioxide films were investigated in [131] using the MD approach. As was the case for fused silica, the peak of the distributions was observed for the *n* = 6 (Figure 6). In the case of the low-energy deposition, the concentration of the rings with a small *n* is higher than in the case of high-energy deposition.

In the final part of this subsection the different results of the atomistic simulations related to the structure of the optical films and film-forming materials are reviewed.

The effects of the energy of the deposited atoms, substrate temperature, and growth template on the structure of the TiO_2_ films were investigated in [153] using the classical MD method with pairwise potential. It was found that the previously nucleated rutile growths in a wider range of temperatures and energies compared to anatase. The increase of the incoming atoms’ energy and substrate temperature leads to the growth of the crystalline phase.

The dependencies of the density of SiO_2_ and coordination number of silicon atoms on the applied pressure were calculated using the MD simulation with a pairwise force field in [101]. Results of the simulation demonstrated that the BKS force field reproduces well the experimental dependence when pressure varies from 0 to 8 GPa.

The growth of the crystalline ZrO_2_ films was simulated in [154]. It was found that the growth of crystalline orientation (001) with a high surface energy and short horizontal period requires a high energy of the incoming atoms. This result agrees with the experiments confirming the increase of the relative preference of this orientation with the growth of the substrate bias voltage. In [155], the MD method was used to simulate the growth of ZnO_x_ films on a crystalline substrate. The effect of the elemental ratio x, energy of the incoming atoms, and fraction of the high-energy atoms to the crystallinity of the growing films were studied. It was found that all mentioned parameters significantly acted on the structure of the film. The influence of the energy distribution of the incoming atoms to the ZrO_2_ films was studied using the MD method in [156]. The results showed that the film density and the nucleation and growth of crystals significantly depend on the fraction of high-energy atoms and the mass of these atoms (zirconium or oxygen). The influence of the substrate temperature on the nucleation of crystals was also revealed.

It has been experimentally shown that the postprocessing of deposited optical films affects their properties. In particular, it was found that thermal annealing leads to a decrease in the optical thickness of the film and the refractive index [157]. The effect of postprocessing on the optical properties of films depends significantly on the method of their deposition [158]. Postprocessing can be modeled at the atomistic level. In [142], the MD method was applied to study the annealing of SiO_2_ films. It was found that the annealing at temperature of 1300 K results in the film density decreasing by about 0.15 g/cm^3^, which corresponds to the reduction of the refractive index of approximately 0.03. Also, the concentration of the nonbridging and threefold coordinated oxygen atoms are reduced up to four times after annealing.

In [33], an approach combining the kMC and MSSD (kMC-MD) methods was developed. The approach was applied to the simulation of the initial steps of ZrO_2_ film growth on Si (1 0 0) surface. The probabilities of the adsorption reactions on the substrate and surface of the growing films were determined from quantum DFT calculations. Furthermore, these probabilities were used in kMC simulations. Also, the force field for the ZrO_2_/SiO_2_/Si film and interface was parametrized based on the DFT calculations of a small number of molecules with appropriate bonding peculiarities.

In [159], the deformation of anatase and amorphous titanium dioxide under uniaxial tension and compression has been studied by atomistic modeling. It was found that, under uniaxial tension, the deformations increase with decreasing grain size. Superplastic deformation took place in anatase with a grain size of 2 nm. It was assumed that this was due to grain boundary sliding and nanosized cavitation.

The MD study of the nucleation and growth of ZnO films on the different substrates was performed in [160]. The formation of the interface between substrate and films was demonstrated. The ZnO deposition into Fe_2_O_3_ and Al_2_O_3_ crystalline substrates results in the formation of the monocrystalline structures. The deposition of ZnO on the silicon dioxide shows less crystallinity.

The multiscale simulation of plasma deposition of TiO_2_ films was performed in [161]. The movement of the sputtered particles was simulated by the direct MC method. The kMC approach was applied for the modeling of the film growth by the condensation and oxidation of the sputtered particles on the substrate. Special attention was paid to the description of the interaction of the charged particles with the previously deposited film layers and substrate. It was found that the suppression of the atom diffusion due to Ti oxidation acts as the formation of the columnar structure. The results of the simulation emphasize the important role of high-energy particles in the formation of the specific features of the deposited film structure.

In [162], the complex simulation of the growth of the TiO_2_ films on the rutile (110) surface was performed. The kinetic Monte Carlo method was used. The authors [162] investigated the effect of different experimental conditions on the film structure, including two kinds of evaporation of the target material—thermal and electron beam—ion beam assisting each other. It was found that evaporation of the target material produces a porous film while the sputtering produces a crystalline structure.

The purity grand of the film-forming materials can have a large influence on the optical properties of thin films [163]. In [164], the MD simulation of the influence of nanoparticles on the properties of deposited silicon dioxide thin films was performed. The interaction of the nanoparticles with film atoms was described by a spherically symmetric potential. The fluctuations of film density near the nanoparticles were revealed. The amplitude of these fluctuations depends significantly on the energy of the deposited atoms.

The growth of Al_2_O_3_ thin films was studied using atom-by-atom MD simulations in [165]. The film structure depends on the deposition parameters such as ion energy, ion fraction in the particle flux, growth temperature and growth template. It was demonstrated that high-energy ions are important for the uninterrupted growth of previously nucleated α-Al_2_O_3_ at low temperatures.

To summarize, the classical atomistic simulation allows calculating the different structural parameters of growing optical films, such as density, porosity, ring distribution, surface roughness, radial distribution function, and concentration of defects. The experimental tendencies, such as changes in the film’s structure with an increase in the deposited atoms energy, are reproduced in the simulations. The problems of the atomistic simulation are the limited dimensions of the simulation clusters, the short trajectories of MD simulations, dependence of the results on the force fields used for the description of the interatomic interactions, and formation of chemical bonds.

### 4.2. Mechanical Properties

The classical atomistic methods are widely used to calculate the mechanical parameters of the deposited films and film-forming materials. Various characteristics of the four TiO_2_ polymorphs were calculated in [119] using the MD simulations. Interatomic interactions were described by the pairwise force field consisting of the Coulomb term, dispersion, and repulsive terms. The force field parameters were fitted to reproduce the crystal structure of rutile, anatase, and brookite, and the experimental values of the rutile elastic constants. It was revealed that the MD simulation reproduces the important mechanical properties of four TiO_2_ polymorphs such as volume compressibility, the thermal expansion coefficient, and enthalpy relationships between polymorphs.

In [166], the structure and bulk moduli of the titanium dioxide polymorphs at both low pressure and high pressure were calculated using the lattice energy minimization with two different force fields. The force field with the variable charge model is more successful in reproducing the bulk moduli of the low-pressure polymorphs. The force field with the fixed charge model somewhat better reproduces the mechanical parameters of the high-pressure phases.

In [167], machine learning was applied to obtain the accurate interatomic potential. The artificial neural networks (ANNs) potential was constructed for titanium dioxide. It was found that the ANN potential reproduces well the lattice parameters, energies, and bulk moduli of TiO_2_ polymorphs. Also, the capability of the potential to predict the properties of high-pressure phases of columbite and baddeleyite was examined.

In [168], the classical MD simulation of the titanium dioxide polymorphs was performed using the pairwise force field. The parameters of the force field were optimized to reproduce the rutile crystal properties. The MD simulation with these parameters reproduces the crystalline structures of the brookite and anatase and mechanical parameters include bulk modulus and volume thermal expansion coefficient.

The MD study of temperature dependence of the TIO_2_ elastic constants was performed in [169]. It was revealed that the elastic modulus decreases gradually with an increase in the temperature from 250 K to 350 K. The relative decrease in this range is about 1%.

In [170], the structure and properties of the physical mixture of In_2_O_3_ and ZrO_2_ were studied using the MD method with different force fields. The lattice parameters of ZrO_2_ were reproduced in the simulation. The relative difference of the experimental and calculated values of bulk modulus strongly depends on the interatomic potentials and varies from 3% to 63%.

The crystalline structural and mechanical properties of ionic and semi-ionic oxides, including the film-forming materials such as SiO_2_ and TiO_2_, were reproduced using the pairwise force field proposed in [102]. The force field was parametrized using the partial ionic charge model with a Morse potential and allows simulating the quenching of melts, glass state, and inorganic crystals. The many-body Tersoff potential was used in [104] to reproduce the structural parameters and cohesive energies for silica polymorphs.

Some results of the simulation of the mechanical properties of fused silica and SiO_2_ films are summarized in Table 5. The elastic moduli for silicon dioxide were calculated using the MD method with a pairwise force field in [101]. It was found that the calculated values of the bulk modulus and Poisson’s ratio are significantly larger than the experimental ones. At the same time Young’s modules and shear modules are reproduced better—the relative difference between the calculated and experimental values is about 10% and 20%, respectively [101]. Also, the changes in the modulus and Poisson’s ratio with growth of the applied pressure up to 6 GPa were qualitatively reproduced in the modeling [101].

In [138], the bulk modulus, Young modulus and Poisson’s ratio for fused silica and SiO_2_ films were calculated using the MD simulation with DESIL force field [80]. It was found that values of all mentioned parameters for the high-energy deposited film are higher than for the low-energy deposited film and fused silica. The calculated values of the Young modulus for low- and high-energy deposited films are close to the experimental ones. A decrease in the energy of the incoming Si atoms leads to a decrease of film density and a decrease of the values of all modules, which is in accordance with the experiment [171,172]. The calculated values of Poisson’s ratio are higher than the experimental ones, 0.19 [173] for SiO_2_ film and 0.17 for fused silica, [172]. At the same time, the excess of the Poisson’s ratio of the film over the Poisson’s ratio of fused quartz agrees with the experiment.

**Table 5:** Calculated values of bulk modulus *K* (GPa), Young modulus *E* (GPa), and Poisson’s ratio β for fused silica and SiO_2_ films. Experimental values noted as “Exp”.

	Fused Silica						SiO_2_ Films	
Ref.	[138]	[101]	[174]	[116]	[175]	Exp. [172]	[138]	Exp.
*K*	25	41 ^1^; 41 ^2^; 54 ^3^	49 ^4^		54 ^6^	37	25 ^7^; 30 ^8^; 35 ^9^	
*E*	50	71 ^1^; 68 ^2^; 81 ^3^		80 ^5^	86 ^6^	73	50 ^7^; 60 ^8^; 70 ^9^	45 ^10^, 74 ^11^
β	0.20	0.21 ^1^; 0.22 ^2^; 0.24 ^3^			0.23 ^6^	0.17	0.23 ^7^; 0.23 ^8^; 0.27 ^9^	0.19 ^12^

^1^ SHIK (Sundarararaman, Huang, Ispas, Kob)-1; ^2^ SHIK-2; ^3^ BKS—pairwise force fields in Ref. [101]. ^4^ three-body potential in Ref. [174]. ^5^ ReaxFF force field in Ref. [116]. ^6^ BKS force field in Ref. [175]. ^7^ E(Si) = 0.1 eV; ^8^ E(Si) = 1 eV; ^9^ E(Si) = 10 eV—energy of the deposited silicon atoms, pairwise DESIL force field [80]. ^10^ low-energy deposition, [132]; ^11^ high-energy deposition, [176]; ^12^ low-energy deposition, [173].

**Figure 7:**
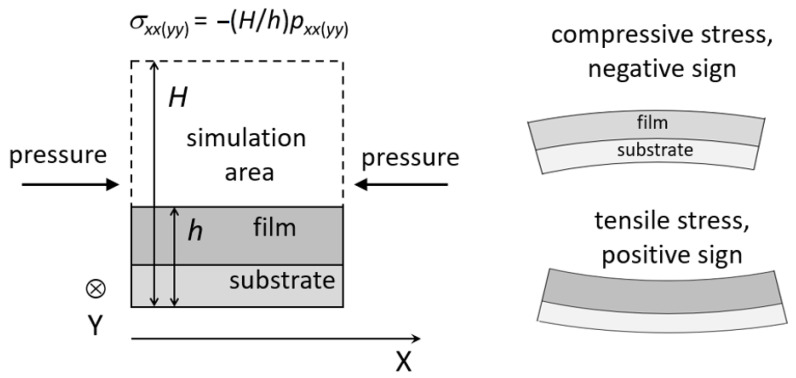
For the calculation of the stresses in the growing films, *σ_xx_*_(*yy*)_ and *p_xx_*_(*yy*)_ are the main components of the stress and pressure tensor, respectively. Adapted from ref. [178].

The atomistic simulation can be used to calculate the stresses that appear during film growth. Stresses are classified as tensile and compressive, having positive and negative sign, respectively (Figure 7, right side) [177]. The main components of the stress tensor are defined using the pressure tensor (Figure 7, left side), which is calculated through the virial [10].

One of the first atomistic simulations of the stress in the growing film was performed in [179], using two-dimensional molecular dynamics. It was revealed that the initial increase of tensile stress is due to the changes of the microstructure of the film when the microcolumnar growth transits to a denser structure with closed micropores. A further decrease in the absolute value of stress is associated with the formation of an ordered structure under the influence of particles with high kinetic energy arriving at the substrate.

In [180], the detailed MD simulation was applied to study the stresses in the thin metal films; some conclusions may be valid for optical films as well. It was found that the energy of the incoming atoms acts dramatically on the stress. The deposition of the particles with energy above 50 eV results in the increase of the disorder in the growing structures and reduction of the tensile stress. After completion of the deposition, the disordered regions restored the crystalline structure, and the stress increased. A reduction of the grain dimensions leads to an increase in the tensile stress.

**Figure 8:**
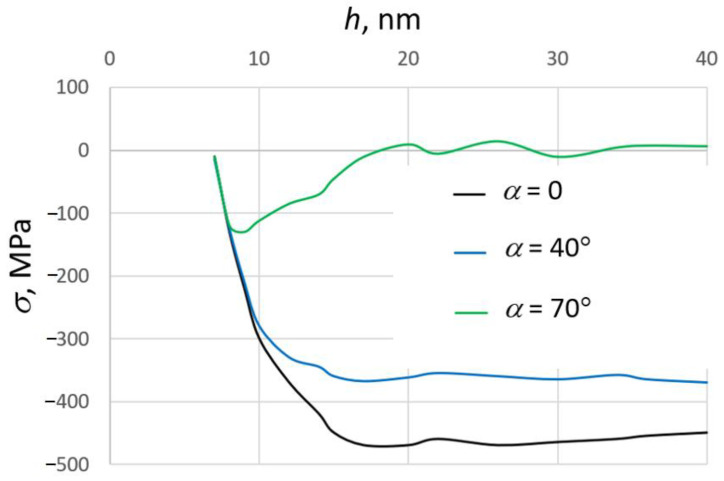
Dependence of the stress on the thickness of the growing silicon dioxide film at different values of the deposition angle α. Deposition energy of silicon atoms is equal to 10 eV, substrate temperature is equal 300 K. Adapted from ref. [178].

The stresses in the growing SiO_2_ films were calculated using the MD method in [178]. The values of the main components of the pressure tensor were averaged over the MD trajectories in the NVT (constant number of particles, volume and temperature) ensemble after the completion of the deposition process. The stress depends essentially on the thickness of the film and the deposition angle α (Figure 8). At small values of α, the stress is compressive. The absolute values of stress at α = 0 and 40° (normal deposition) are in the interval of the experimental data [128,177]. The increase of the deposition angle up to 70° leads to a significant decrease in the stress value and even changes the stress type from compressive to tensile. This is due to the formation of the porous structure at these conditions. A change of the sign of the stress for depositions on large-angle films with increasing thickness was observed experimentally [181,182].

In [183], the MD simulation of nanoindentation of the amorphous SiO_2_ film on the monocrystalline silicon was performed. It was found that at the same indentation depth, the values of modulus decrease with the growth of the film’s thickness due to decrease of the silicon substrate effect. The densification of the film near the nanoindentation area was attributed to the rotation and deformation of SiO4 tetrahedrons.

The mechanical loss in optical coatings in one of the factors limited the accuracy of the high-precision gravitational wave detectors in LIGO (Laser Interferometer Gravitational-Wave Observatory) project. These losses were calculated for the amorphous pure and doped silica [184] and amorphous tantala and titania-doped tantala [185] using the two-wells model based on the atomistic simulation of the film structure. The parameters of the potentials, used in the MD simulations, were fitted to reproduce the experimental RDF and elastic constant. The low-temperature peaks in the loss were reproduced in this model.

Thus, atomistic modeling with various types of force fields is widely used to calculate the mechanical parameters of the amorphous phase of film optical materials. These force fields are parameterized to reproduce the geometry of cells and some other parameters of crystalline polymorphs. At the same time, these force fields have not yet found wide application for calculating the mechanical parameters of deposited films. This problem is relevant, since the mechanical parameters of the films and the possibility of their application depend significantly on the conditions of film production.

### 4.3. Kinetics, Thermodynamics, and Other Properties

The thermal conductivity (TC) of thin films and film-forming materials is an important kinetic parameter, which was studied by the atomistic simulation. The temperature dependence of the TC of amorphous SiO_2_ was studied using the molecular dynamics method in [186]. Two thermal transport mechanisms were revealed. The first is temperature independent and is related to short-length scale behavior. The conductivity by the second mechanism depends on the temperature and relates to long-length scale behavior. The temperature dependence of TC for quartz was calculated in [187] using the MD method. The calculated values of TC were in reasonable agreement with the experiment. It was found that the distortion of the SiO_4_ tetrahedra decreases the TC. The contributions of the different vibrational bands to the thermal conductivity were identified in [187] using the heat current autocorrelation function.

The TC of the different film-forming materials was studied using the nonequilibrium molecular dynamics (NEMD) method in [188,189,190,191]. The TC values of crystalline and amorphous SiO_2_ thin films were calculated in [188]. The temperature varies from 100 to 700 K. It was found that the temperature dependence of TC of amorphous thin films is similar to that for bulk materials [188]. The TC of amorphous nanoporous silica was studied in [189]. The pore diameter and porosity varied from 12 to 25 Å and from 10% to 35%, respectively. It was found that TC depends on the porosity and is independent of pore size [189]. In [190], thermal transport in a-HfO_2_ was studied. It was found that the TC value grows with an increase in the temperature and thermal transport is sensitive to the dimensions of the systems. In [191], the TC of a-SiO_2_ thin films was investigated. It was found that the TC is independent of the temperature in the interval from −55 °C to 150 °C. The TC of a-SiO_2_ is less sensitive to defects than TC of c-SiO_2_ thin films. In [192], the TC of multilayer dielectric films consisting of SiO_2_ and Al_2_O_3_ was studied using the MD approach. It was found that the TC of multilayer structures noticeably decreases compared to that of the bulk dielectrics. The thickness dependence of TC was observed for the crystalline multilayer but not in the amorphous one. The MD simulation was used in [193] to study the TC of TiO_2_/ZnO nanofilm interface. It was found that the increase of the temperature from 300 K to 600 K results in a decrease of TC. A similar result was obtained by increasing the film thickness from 1.8 to 5 nm.

The MD simulation with a BKS force field was used to calculate the specific heat of amorphous silica in the frame of the harmonic approximation [194]. The velocity autocorrelation function and vibrational density of states (VDOS) were calculated. It was found that the harmonic approximation is valid if the temperature is below 300 K. The calculated value of the specific heat at temperatures below 50 K is two times lower than the experimental value. At the same time, in the temperature interval from 200 K to the glass transition temperature, 1450 K as the calculated value of specific heat agrees with the experiment.

The VDOS of amorphous silica was also calculated in [101]. It was found that the BKS potential predicts well the splitting of the high-frequency stretching modes. At the same time, the intermediate interval of the frequencies in the peaks near 400 cm^−1^ and 750 cm^−1^ are absent in the VDOS calculated using the BKS. Nevertheless, the authors of [101] conclude that the new simple pairwise potential parametrized as suggested in [101] reproduces experimental VDOS more accurately than the potential with the more complicated functional form.

As mentioned in the previous section, in [168], the thermal expansion coefficient of titanium dioxide was reproduced in the MD simulation with a pairwise force field with parameters, fitting the rutile structure.

The values of the surface energy of SiO_2_ nanoclusters were calculated in [195] using the MD method. It was revealed that the surface energy is essentially less than the experimental value of the surface energy of amorphous silica and depends nonmonotonically on temperature.

In [196], the atomistic moment method in statistical (SMM) [197] dynamics was applied to investigate the thermodynamic properties of ZrO_2_ thin films. It was revealed that the thermal expansion coefficient decreases with an increase in pressure and grows with an increase in the temperature and thickness. The thermodynamic quantities for ZrO_2_ thin films with more than twenty layers are in good agreement with the experimental value of bulk ZrO_2_.

Thus, the thermal conductivity of film-forming materials is the subject of research by atomistic modeling methods in many works. This parameter determines the distribution of heat fluxes in films during their heating and affects the processes associated with heat fluxes, for example, thermally induced stresses. Such stresses arising at the interface between films of different compositions can damage the structure of a multilayer optical coating. In this regard, the study of the dependence of thermal conductivity on optical films on the deposition conditions is an important task of atomistic modeling.

### 4.4. Quantum Simulation of Optical, Electronic, and Structural Properties of Film-Forming Materials

The optical and electronic parameters of optical thin films and coatings are of key importance for their applications. These properties depend on the film structure and deposition conditions. For the calculation of the optical and electronic parameters, the ab initio quantum methods should be used. Also, QM allows calculating the geometry and properties of the point defects and related states in the gap. As is mentioned in Section 3.1, these are very time-consuming methods compared with classical atomistic methods, so the simulation clusters as a rule include no more than several hundred atoms. For this reason, QMs are not applied directly to PVD simulations but are used to model film-forming materials.

The quantum molecular dynamics, based on the DFT method with local density approximation (LDA) and Vanderbilt ultra-soft pseudopotentials [198] implemented into the VASP [36] program, was applied in [199] to study the properties of amorphous TiO_2_. The simulations were performed with two clusters, including 96 and 192 atoms. The melting-quenching procedure was applied to obtain the amorphous state. The densities were calculated in the *NPT* (constant number of particles, pressure and temperature) ensemble and were equal to 3.59 g/cm^3^ and 3.73 g/cm^3^ for 96 and 192-atom clusters, respectively. The last value was closer to the experimental density of amorphous TiO_2_, 3.59 g/cm^3^ [97]. It was found that structural parameters (peak position of the RDF, coordination numbers of Ti and O atoms, angle distributions) of the structures obtained this way correspond to the experimental ones. The valence and conduction tail states, occurring in the amorphous TiO_2_, were attributed to the disorder in the position of oxygen atoms and overcoordinated Ti atoms, respectively.

The electronic structure of titanium dioxide rutile with oxygen vacancies was studied in [200]. The simulations were performed using the Kohn–Sham DFT approach [201] with the exchange-correlation function of the generalized gradient approximation in the spin-polarized variant. The Coulomb interaction between 3d electrons of Ti atoms was considered in the frame of the Hubbard model. The simulation cell consists of 96 atoms. The calculated values of the band gap vary from 1.9 eV to 2.2 eV, which are essentially lower than the experimental value 3.0–3.2 eV [202]. This difference was explained by the systematical underestimation of band gap value by the DFT methods. It was found that the capture of both an electron and a hole in the oxygen vacancy is accompanied by energy gain. So, these defects in the TiO_2_ serve as electron and hole trap that can significantly affect the optical properties of titanium dioxide.

In [105], the structural and electronic properties of amorphous titanium dioxide were studied by classical and quantum–atomistic methods. Based on the results of quantum DFT simulation, a model for the structure of titanium dioxide is proposed. The model reproduces the structural parameters of the deposited amorphous layers, such as coordination numbers and RDF peak positions. The electronic properties were also studied. It was found that the band gap decreases with an increase in the density of the amorphous state. In addition, the results of the classical MD simulation of amorphous titanium dioxide were validated against the quantum DFT approach in [105]. The RDF, structure factor, cell density, and coordination numbers obtained from MD and DFT simulations were found to be close to one another. The authors of [105] assumed that the correlation of electronic parameters with disorder in the amorphous phase, including the occurring of the under- and overcoordinated atoms, can serve as a basis for estimation of the optical quality of deposited films.

The quantum DFT method was applied in [77] to study the properties of amorphous TiO_2_ using the VASP program [203]. The simulation was performed in two stages: relaxation of the atomic positions and calculation of the real and imaginary part of the frequency-dependent dielectric function. Good agreement between the calculated and experimental values of the refractive index was achieved for wavelengths greater than about 1000 nm. For shorter wavelengths, the experimental value of the refractive index somewhat exceeded the calculated value.

In [204] and in [205], the ab-initio molecular dynamics approach was applied to the calculation of the different properties of the polymorphs of ZrO_2_ and HfO_2_. Methods for obtaining the structure of the amorphous ZrO_2_ were also discussed. It was found that the dielectric constant of amorphous ZrO_2_ is comparable to that of the monoclinic phase. The calculated value of the band gap of 3.4 eV was less than experimental values 5.3 and 5.1 eV measured in thin films of a-ZrO_2_ [206]. This difference was attributed to the tendency of the DFT-LDA method to underestimate the band gap of insulators.

In [207] the ab initio, MD modeling of liquid and amorphous SiO_2_ was performed. The simulation cluster consists of 192 atoms and the length of the MD trajectory was 20 ps. The initial states for the simulation were obtained by the quenching of random configurations of Si and O atoms. The possibility of the formation of two-membered rings, indicating the internal stress in the structure, was studied.

In [208], the quantum MD simulation was used to obtain the amorphous silica from the SiO_2_ crystal. Two different amorphous states were obtained from the melts stabilized at two temperature intervals: 3000–4000 K and 5000–6000 K. The first state was characterized by a structure similar to the initial crystal and fused silica without point defects. The second state was less regular and contained point defects of different types, including 2-Bridging Oxygen Center. This model can be used for the description of the structure of amorphous silicon dioxide films obtained by the high-energy ion-beam deposition process.

The modeling of amorphous states of SiO_2_, HfO_2,_ and ZrO_2_ was performed in [209] using the DFT-based quantum molecular dynamics method. The amorphous states were obtained by melting SiO_2_, HfO_2,_ and ZrO_2_ crystals in the *NPT* ensemble. The density of the electronic states was calculated for the crystalline and amorphous phases. It was found that the bang gap of amorphous zirconium dioxide and hafnium dioxide was clear from the defect states. In ZrO_2_ and HfO_2_, with an increase in the melt temperature, a sharp decrease in density was observed accompanied by a decrease in the coordination number of atoms.

The review of the quantum simulation of SiO_2_, HfO_2_ and ZrO_2_ film-forming oxides presented in the Introduction of the Ref. [209]. The conclusion from this review is that the structural characteristics of the amorphous phase of film-forming materials and their optical and electronic parameters significantly depend on the simulation method. Thus, validation of the level of the QM used in modeling is a necessary step in the simulation procedure.

The QM also can be applied for the simulation of the film surface. In [210,211], the model of the hydroxylated surface of a-SiO_2_ was developed and characterized using the periodic DFT calculations. The detailed comparison of the calculated and experimental parameters characterizing this surface was performed in [210]. The surface construction method can be useful in modeling the interaction of the film surface with small molecules in the gas phase. The models of the amorphous silica and its surface were discussed in the recent review [212]. The authors of [212] showed that the quantum DFT can be used as an accurate method for the investigation of the surface reactions.

To summarize, it can be concluded that the QM are widely used in the simulation of dielectric film-forming materials. The different methods of the generation of the amorphous phase were developed, including the melting-quenching procedure. The advantage of the quantum MD method over the classical MD method when implementing this procedure lies in a more adequate description of the breaking, switching, and formation of chemical bonds. It was demonstrated that QM allows obtaining structures with parameters that are consistent with experiment data. However, the most interesting electronic and optical properties depend significantly on the level of simulation.

### 4.5. Glancing Angle Deposition

**Figure 9:**
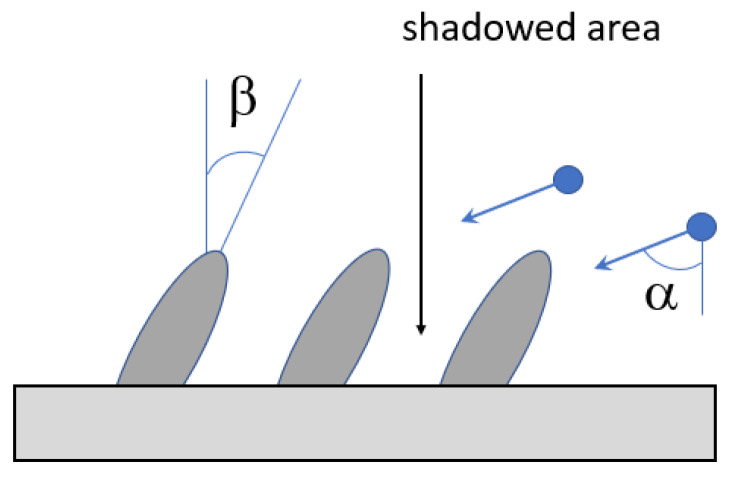
Geometry of the glancing angle deposition. The deposition and tilt angles are noted as α and β, respectively.

The deposition under the large angle results in the formation of the separated nanostructures having different shapes and dimensions [5,213]. These structures grow due to the appearance of the shadowed regions behind the nuclei of the growing film on the substrate (Figure 9) [214,215]. Incoming atoms do not penetrate into these regions, which leads to the formation of separated structures.

This method of film production is called GLAD (glancing angle deposition). The GLAD films are characterized by the anisotropy of the structure and properties, high porosity, and a low refractive index [215].

The ballistic approach was used initially for the modeling of the GLAD [216]. In this approach, the film is represented as a set of the cell on the substrate surface. The occupation of the cell by the deposited atoms is described using the Monte Carlo method. The ballistic approach was used in a 3D-ballistic simulator [217] for modeling the growth of the GLAD films. It was found that the deposition under the glancing angle results in a reduction of the film density by 2.5 times compared with case of the normal deposition. The study of the dependency of the tilt angle on the deposition angle in the frame of the ballistic approach was performed in [218]. Based on the simulation results, the model predicting this dependency in the wide range of the deposition angles was proposed in [218]. The simple rules for the estimation of the tilt angle from the deposition angle were derived taking into account the geometry of the glancing angle deposition and are known as the “tangent rule” [219] and the “cosine rule” [214]:

2tgβ = tgα(2)

*β* = *α* − arcsin((1 − cosα)/2) (3)

Other more complex empirical expressions were proposed in [220,221,222]. All these rules state that the tilt angle is less than the deposition angle that corresponds to the experimental data [223]. At the same time, the tilt angle also depends on the material properties, so the simple geometric approaches are limited in the description of the relation between deposition angle and tilt angle.

The ballistic approach is also used for the simulation of the GLAD films taking into account the rotating of the substrate, divergence of the flow of the deposited atoms, and variation of the deposition angle [224]. It was found that the results of these simulations for the silicon films agree with the experiment. The 3D MC simulator was elaborated and applied to study the structure of GLAD films in [225]. The nanostructures of different shapes, including zigzag, helix, and cylinder, were reproduced in the simulation. It was found that the effective surface of the film depends insignificantly on the columnar shapes and achieves a maximum at a deposition angle of 70°. The 3D MC simulator ADEPT (Atomistic simulator for thin film DEPosition in three dimensions) [31] was used in [226] to simulate the growth process. It was found that in order to obtain agreement between the calculated and experimental structural properties of the film, it is necessary to include high-energy particles in the simulation. The MC modeling was used in [227] to study the structure of ZrO_2_ thin films. The simulation revealed two deposition modes affecting the film structure: shading-dominated growth at low temperatures and diffusion-dominated growth at high temperatures and a low deposition rate.

The grid effect in on-lattice simulations of GLAD films was studied in [224]. The method for reducing this effect was developed and applied to the silicon films. It was demonstrated that on-lattice simulations with this method generate structures consistent with the experiment.

The MD was also used to simulate the growth of GLAD films. In [228], the study of the GLAD film structure on the deposition conditions was performed using the two-dimensional MD simulation. It was found that the tangent rule fits the relationship between deposition angle α and tilt angle well when the α < 60°. Also, the tilt angle β decreases with an increase in the energy of the incoming atoms due to an increase in their surface mobility. The MD simulation of aluminum films having columnar structure was performed in [229]. It was revealed that the templates on the substrate act significantly on the shadow effect and columnar structures. The growth of the Cu layers at a deposition angle varying from 50° to 85° was studied in [230] using the MD simulation. The formation of the columnar structure was observed when the deposition angle exceeds 80°.

The structural properties of SiO_2_ GLAD films were studied using the MD method in [231]. It was found that the separated nanostructures are formed at high- and low-energy depositions at large angles. The increase of the substrate temperature and annealing of the deposited films lead to the merging of the slanted columns. The MD simulation of the formation of high-energy SiO_2_ GLAD films with alternation of the deposition angle was performed in [232]. At a fast alternation, the growth of treelike structures was demonstrated, while the low alternation results in the formation of chevron-like structures. The substrate rotation leads to the formation of the helical structure.

The dependence of stress values in SiO_2_ films on their thickness was studied in [178] using the MD method. It was found that in GLAD films, the stress is several times lower than in the normally deposited films. Additionally, the stress in GLAD films varies from compressive to tensile with the growth of the film’s thickness [178].

The growth of high-energy TiO_2_ GLAD films was simulated in [95] using the classical MD method. The deposition angle varied from 50° to 70°. The geometric parameters of the elongated pores separating the slanted columns were determined according to the method proposed in [78]. The components of the refractive index tensor were calculated based on the effective medium approach [233]. The good agreement of the calculated and experimental values was observed at relatively small deposition angles [95]. The MD and kMC methods were applied in [129] for the simulation of the deposition of the GLAD titanium dioxide films. The parameters describing the anisotropy of the structure and refractive index were calculated using the effective medium approach [233]. It was found that the MD results agree better with the experiments than the kMC results. In [234], the different aspects of the atomistic simulation of thin films growth are investigated, including the influence of the force field and dimensions of the simulation area on the structure of the deposited films. It was found that the value of the film’s density depends essentially on the force field. At the same time, the common features of the density profiles were observed. The difference Δn of the main components of the refractive index was calculated for the TiO_2_ GLAD films [234]. It was found that the Δn values depend significantly on the dimension of the simulation cluster.

**Figure 10:**
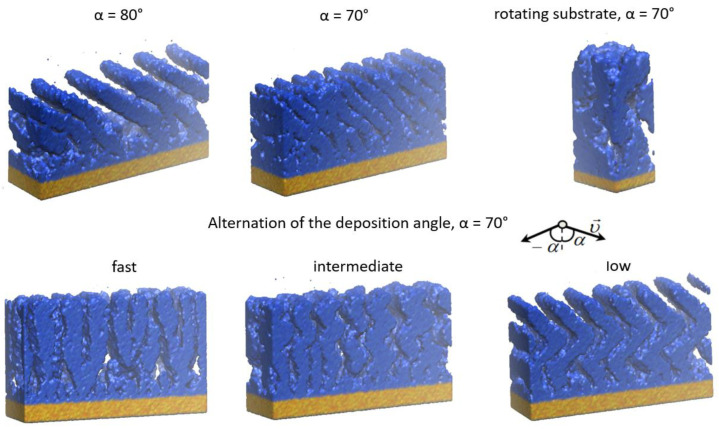
GLAD structure obtained in MD simulation, α is the deposition angle. Energy of the incoming atoms is equal to 10 eV, substrate temperature T = 300 K. Adapted from ref. [232].

The results of the MD simulation of the typical SiO_2_ GLAD structure, deposited at high energy of incoming atoms, are shown in Figure 10. The slanted columns are formed at the condition of the constant direction of the incoming atoms flow (Figure 10, top row). The tilt angle increases with the increase in the deposition angle from 70° to 80°. The rotation of the substrate results in the formation of the helical-like structure. In the condition of the alternation of the deposition angle, the shape of the growing structures depends on the rate of the alternation (Figure 10, bottom row). Fast and low alternations result in the growth of three-like and zig-zag-like structures, respectively. The alternation with the intermediate rate leads to the formation of films having mixed shapes.

Thus, an atomistic simulation reproduces the general trend toward a decrease in film density with increasing deposition angles. Just as in the experiment, starting from a certain value of the deposition angle, the growth of separated nanostructures is observed. The simulation reproduces various types of these structures depending on the deposition conditions. The use of MD, MC, and kMC methods makes it possible to study the formation of GLAD films taking into account the atomic structure of the film-forming material. Also, the anisotropy of the GLAD structure can be quantified by combining the atomistic approach and the effective medium approach.

## 5. Conclusions and Future Directions

At present, a wide range of atomistic methods are used to simulate the physical vapor deposition of thin optical films and study their properties. Approaches based on the Monte Carlo method are used to simulate the processes in a vacuum chamber: the sputtering of particles from a target, their transport through the chamber to the substrate, and the formation of film layers on the substrate surface. Molecular dynamics are mainly used to model the formation of film layers. Quantum methods are used to calculate the structural, electronic, and optical parameters of film-forming materials and to parametrize force fields for classical atomistic methods.

The current level of modeling makes it possible to reproduce important characteristics of film-forming materials, such as the structure and mechanical characteristics of their polymorphs, thermal conductivity, specific heat and other thermodynamic parameters, gap width, state generation in the gap due to disorder in the amorphous phase and the formation of point defects. Many experimental dependencies of film properties on PVD conditions are reproduced in atomistic simulations: an increase in density, surface roughness, porosity, and stresses with an increase in the energy of incident atoms; the formation of porous and anisotropic structures with an increase in the deposition angle (GLAD films); the dependence of the refractive index on deposition conditions; and mechanical parameters of films.

The directions of further development of atomistic modeling of optical thin films strongly depend on the growth of computing power. In classical methods, the most time-consuming part of the simulation is associated with the calculation of the long-range electrostatic interaction between atoms. Due to these interactions, the number of atoms in full-atomistic MD simulations of optical thin films is limited to a few million even with GPU parallel computing, while for single-component materials such as metal, this number can exceed tens of millions. An increase in the number of atoms in clusters by several times will make it possible for the first time to carry out an atomistic simulation of optical coatings consisting of several layers with different properties.

In the last decade, the new type of machine-learned force fields, parametrized with high accuracy by a large number of quantum DFT calculations, has been actively developed. The accuracy of these force fields is close to that of quantum computing, but their numerical efficiency is much higher. Up until today, these force fields have been used for the calculations of bulk properties, and in perspective can be applied for detailed modeling of the formation of chemical bonds on the surface of a film and substrate, oxidation of incident atoms, and modeling of defects in transition layers between films of different compositions.

Despite the development of these force fields, the problem of quantum modeling of film growth remains relevant, especially in the case of high-energy deposition, when atoms and ions with energies exceeding several eV interact with the substrate and films. These high-energy particles can create nonequilibrium configurations that affect film properties. Also, the calculation of the parameters of the valence and conduction bands in the transition region between layers of different compositions is important for evaluating the electronic and optical properties of multilayer coatings.

## Figures and Tables

**Table 1:** Brief characterization of force fields used for the classical simulation of thin optical films and film-forming materials.

**Pairwise Force Fields**	
Method of parametrization	Reproduction of experimental structural and mechanical properties of film-forming materials
Advantages	High computational efficiency; cluster sizes up to tens of nanometers and number of atoms up to millions
Disadvantages	Only stoichiometric materials, limitations in the description of defects and the formation of chemical bonds and surface effects
Possible application area	Modeling the structure of growing films (density profiles, porosity, surface roughness, etc.), including the formation of nanostructured films with high porosity. Calculation of mechanical parameters of films
**Many-body Force Fields**	
Method of parametrization	Reproducing the QM calculation of atomistic clusters and molecules, structural, thermodynamic and mechanical parameters of polymorphs of films forming materials
Advantages	Description of the film structure with an accuracy close to the QM level
Disadvantages	Relatively low computational efficiency; clusters dimensions up several nanometers and number of atoms up to tens of thousands
Possible application area	Simulation of structure of non-stoichiometric materials, all types of point defects, surface effects, structure of transition regions between layers of different compounds. Calculation of mechanical parameters of films.

**Table 4:** Relative pore volume in silicon dioxide films *f*(*R*), *R* (nm) is the effective dimension of the pore, *E* (eV) is the energy of the incoming Si atoms [144]. Substrate temperature is equal to 300 K.

*R*	0.05	0.1	0.2	0.3	0.4
*f*(*R*), *E* = 0.1	0.14	0.071	0.023	0.010	0.004
*f*(*R*), *E* = 1.0	0.075	0.015	0.001	0.001	<10^−5^
*f*(*R*), *E* = 10	0.038	0.004	<10^−5^	<10^−5^	<10^−5^

## Abbreviations

GLAD	glancing angle deposition
PVD	physical vapor deposition
MD	molecular dynamics
MC	Monte Carlo
kMC	kinetic Monte Carlo
QM	quantum methods
GROMACS	GROningen MAchine for Chemical Simulations
LAMMPS	Large-scale Atomic/Molecular Massively Parallel Simulator
NAMD	NAnoscale Molecular Dynamics
NASCAM	NAnoSCAle Modeling
VASP	Vienna Ab initio Simulation Package
GAMESS	General Atomic and Molecular Electronic Structure System
TRIM	TRansport of Ions in Matter
SRIM	The Stopping and Range of Ions in Matter
EAM	Embedded Atom Model
ZBL	Ziegler, Biersack and Litt- mark
MEAM	Modified Embedded Atom Method
DFT	Density Functional Theory
SIMTRA	Simulation of Metal TRAnsport
RSD2013	Reactive Sputter Deposition
PIC/MCC	particle-in-cell/Monte Carlo
REaxFF	REActive Force-Field
PME	Particle Mesh Ewald
BKS	Beest Kramer van Santen
DESIL	DEposited SILica
MA	Matsui and Akaogi
RDF	radial distribution function
ANNs	artificial neural networks
SHIK	Sundarararaman, Huang, Ispas, Kob
NVT	constant number of particles, volume and temperature thermodynamic ensemble
NPT	constant number of particles, pressure and temperature) ensemble
LIGO	Laser Interferometer Gravitational-Wave Observatory
TC	thermal conductivity
NEMD	nonequilibrium molecular dynamics
VDOS	vibrational density of states
LDA	local density approximation
GLAD	glancing angle deposition
